# Optimized multi-echo gradient echo MRI of the lumbosacral spinal cord to investigate atrophy, lesions, and disability in relapsing-remitting multiple sclerosis

**DOI:** 10.1162/IMAG.a.1410

**Published:** 2026-11-10

**Authors:** Gabriella L. Dunay, Xinyu Zhang, Funminiyi Adepegba, Anna Combes, Alicia E. Cronin, Lipika Narisetti, Grace Sweeney, Kritin Vasamreddy, Seth Stubblefield, Colin D. McKnight, Logan Prock, Delaney Houston, Atlee A. Witt, Megan McGrath, Simon Vandekar, Subramaniam Sriram, Seth A. Smith, Kristin P. O’Grady

**Affiliations:** Department of Biomedical Engineering, Vanderbilt University, Nashville, TN, United States; Department of Biostatistics, Vanderbilt University Medical Center, Nashville, TN, United States; Vanderbilt University Institute of Imaging Science, Vanderbilt University Medical Center, Nashville, TN, United States; NMR Research Unit, Department of Neuroinflammation, Queen Square Multiple Sclerosis Centre, UCL Queen Square Institute of Neurology, University College London, London, United Kingdom; Department of Radiology and Radiological Sciences, Vanderbilt University Medical Center, Nashville, TN, United States; Vanderbilt University School of Medicine, Nashville, TN, United States; Department of Neurology, Vanderbilt University Medical Center, Nashville, TN, United States

**Keywords:** atrophy, biological variability, lumbosacral spinal cord, magnetic resonance imaging, morphometry, multiple sclerosis

## Abstract

The lumbosacral enlargement (LSE) of the spinal cord is understudied in people with multiple sclerosis (pwMS), and there is not a consensus on the impact of age, sex, height, or weight on MRI-derived morphometrics in this region. The LSE is relevant to study in pwMS as it is involved in the control of motor and sensory functions of the lower limbs and autonomic functions which are commonly affected in MS. This study aimed to characterize biological variability of morphometric measures of the LSE cord and gray matter in healthy controls (HCs), compare these measures between HCs and people with relapsing-remitting MS (pwRRMS), and assess correlations between morphometrics and lower-extremity disability and bladder symptoms in pwRRMS. An optimized T2*-weighted, axial multi-echo gradient echo anatomical MRI scan was acquired at 3T, images were automatically segmented, and whole-cord and gray matter cross-sectional areas for the lumbosacral spinal levels of 60 HCs and 42 pwRRMS were computed. We observed no significant main effects of age, height, weight, or sex on LSE whole-cord or gray matter cross-sectional areas (CSA) in HCs, but there were spinal level interactions with age and weight. We detected whole-cord and gray matter atrophy in the LSE of pwRRMS compared to HCs, and the effects of disease status on these measures were partially mitigated when controlling for MS lesion to cord CSA ratio within the LSE. There were no significant associations between LSE cross-sectional area measures and sensorimotor function, bladder symptoms, or clinical disability scores. However, vibration sensation in the great toe and Expanded Disability Status Scale (EDSS) scores were significantly associated with lesion to cord CSA ratio. Overall, this study found that there is little variability linked to age, sex, height, or weight in cross-sectional area measures for the healthy lumbosacral spinal cord, and lesions and atrophy were both detected in these spinal levels for a pwRRMS cohort. Although there were no associations between atrophy and symptoms, potentially due to the relatively low level of clinical disability in this cohort, these findings support the inclusion of the LSE in future imaging studies that aim to probe the full extent of spinal cord pathology in MS.

## 1 Introduction

Multiple sclerosis (MS) is a chronic autoimmune, neurological disorder characterized by inflammatory demyelination and irreversible axonal loss in the central nervous system (McGinley et al., 2021). Magnetic resonance imaging (MRI) is a critical clinical tool for both diagnosing MS and routinely monitoring disease activity, and it is widely used in research to deepen understanding of complex demyelinating and neurodegenerative processes in MS (Muccilli et al., 2018; Sastre-Garriga et al., 2020). The spinal cord (SC) is a compact structure with tightly organized gray and white matter pathways that mediate essential motor and sensory functions, making it a region of particular interest for investigating the structural and functional impacts of MS pathology (Muccilli et al., 2018). Cervical SC atrophy has been established as a biomarker for disability in MS, with prognostic value across all stages and clinical phenotypes (Sastre-Garriga et al., 2020).

While the cervical SC has been extensively studied in MS (Casserly et al., 2018), the lumbosacral SC has received comparatively less attention despite its important functional roles. The lack of advanced MRI investigations focused on the lower SC anatomy is due in part to technical challenges, including the small size (Stroman et al., 2014), participant motion and physiological motion from respiration, heartbeat, and cerebrospinal fluid pulsation (Muccilli et al., 2018; Stroman et al., 2014), and spatially- and temporally-varying magnetic field inhomogeneities that arise from susceptibility differences at the interfaces between bone, soft tissue, and air (Stroman et al., 2014). The proximity to the lungs of thoracic and lumbosacral SC segments further exacerbates field inhomogeneity fluctuations compared to the cervical SC segments (Dehghani et al., 2020). The lumbosacral enlargement (LSE), located at the L2-S2 spinal levels, where spinal level refers to the neurological SC level, and identified by its increased lateral and anteroposterior dimensions (Diaz & Morales, 2016), contains a dense network of neurons involved in the control of motor and sensory functions of the lower limbs and autonomic functions (Toossi et al., 2021). It plays a key role in regulating locomotion, micturition, bowel, reproductive functions, and sensory processing from the legs, feet, anal, and genital regions (Guertin, 2013). These lower body functions are frequently affected in MS. Bladder dysfunction and fecal incontinence affect approximately 75% (Nortvedt et al., 2007) and up to 66% (Thiruppathy & Preziosi, 2014) of people with MS (pwMS), respectively. After 10 years of disease duration, 59% of pwMS experience mild or greater gait disability (Kister et al., 2013).

Despite its clinical relevance to lower extremity and autonomic symptoms frequently experienced by pwMS, the LSE remains understudied in MS. In MS, gray matter (GM) atrophy is thought to start earlier than white matter (WM) atrophy and has strong correlations with clinical disability (Keegan et al., 2024; Schlaeger et al., 2014), so it is important to develop MRI methods that provide adequate intra-cord tissue contrast and image resolution for separate quantification of GM and WM tissue metrics in addition to whole-cord volume. Recently, high-resolution T2*-weighted multi-echo gradient echo (mFFE) sequences have been optimized for the lumbosacral SC using 3 Tesla (3T) MRI (Büeler et al., 2022; O’Grady et al., 2023). The optimized mFFE MRI sequence for the lumbosacral SC enhances GM to WM contrast and lesion sensitivity, enabling better quantitative assessment of tissue morphometry in the LSE. This, in turn, allows for the opportunity to examine tissue atrophy and lesion presence in MS (White et al., 2011), and may offer additional insight into disease progression and relevant symptoms.

Before comparing SC cross-sectional area (CSA) differences between healthy controls (HCs) and pwMS in the LSE, it is important to evaluate biological variability in the imaging outcomes. Differentiating disease-related changes from natural variation among individuals enables more accurate interpretations of MRI-derived metrics and assessments of disease effects. Prior studies have investigated potential normalization strategies for cervical SC MRI measurements, including normalization of CSA measures to intracranial volume (Healy et al., 2011; Kesenheimer et al., 2021), body mass index (Healy et al., 2011), body surface area (Healy et al., 2011), spinal canal dimensions (Kesenheimer et al., 2021; Papinutto et al., 2019), brain volumes (Kesenheimer et al., 2021), and/or head size (Papinutto et al., 2019). However, these normalization strategies have not been standardized in the field, and they have shown limited associations with SC areas at the LSE (Kesenheimer et al., 2021) likely due to the region’s inherently high anatomical variability compared to the upper spinal levels (Kesenheimer et al., 2021; Schlaeger et al., 2015). Additionally, effects of biological variables, including sex, age, height, and weight, on SC morphometrics, have been investigated, but the findings have varied between studies and between SC regions (Cook et al., 2024; Kesenheimer et al., 2021; Labounek et al., 2025; Papinutto et al., 2015, 2019; Ulbrich et al., 2014; Valošek et al., 2024; Yiannakas et al., 2019). These prior investigations into normalization strategies and biological sources of variability for SC morphometry have predominantly focused on the cervical region, with far fewer investigations in the thoracic SC and LSE.

In this study, we evaluated the effects of biological variables on MRI-derived measures of LSE SC and GM CSA using an optimized T2*-weighted mFFE sequence applied in HCs. We then compared SC CSA, GM CSA, and GM:Cord CSA ratio between HCs and people with relapsing-remitting MS (pwRRMS) to assess sensitivity to cord atrophy in the LSE and evaluated the contribution of the Lesion:Cord CSA ratio to these group differences. Finally, we investigated the relationships between LSE morphometry and lesion measures and sensorimotor and clinical disability measures in pwRRMS.

## 2 Methods

### 2.1 Ethical approval and inclusion criteria

All experimental protocols were approved by the Vanderbilt Institutional Review Board (Protocol #111087, enrollment spanning 2020-2024). All methods were conducted in accordance with relevant ethical guidelines and regulations. Inclusion criteria for pwRRMS were age ≥18 years, an Expanded Disability Status Scale (EDSS) score ≤6.5, a diagnosis of relapsing-remitting MS (RRMS), and no contraindications to 3T MRI. Inclusion criteria for HCs were age ≥18 years, no history of SC injury, neurodegenerative, or demyelinating conditions, and no contraindications to 3T MRI.

### 2.2 Study participants

After written informed consent, 60 individual HCs (40F/20M) and 42 individual pwRRMS (32F/10M) completed SC MRI, and a subset of these participants (18 HCs, 37 pwRRMS) completed sensorimotor testing. MRI data from a second visit were also included for 15 of the 60 HCs in this study. Participant demographics and disease duration (Median = 7.07 yrs, Range = [0.170, 33.9] yrs) are summarized in Table 1, and sensorimotor/disability scores are summarized in Table 2. The cohort of pwRRMS had relatively low clinical disability: 88% of those with reported EDSS scores had EDSS < 4, and 86.5% demonstrated 25-foot walk times of less than 6.6 seconds, which has been reported to correspond with EDSS ≤ 4 (Kalinowski et al., 2022). Before forming this final cohort of participants for analysis, 10 HCs and 1 pwRRMS were excluded due to severe motion artifacts on the mFFE images.

**Table 1.** Biological data for healthy controls and people with multiple sclerosis.

	Healthy controls (HC)	Multiple sclerosis (MS)	p-value (HC vs. MS)
Sex			
Female	40	32	0.2988
Male	20	10	Chi-squared
Age			
Mean ± SD	35.4 ± 12.1	40.6 ± 11.3	0.0263
Median [min, max]	33.0 [19.0, 74.0]	39.3 [21.0, 64.1]	
Height			
Mean ± SD	66.4 ± 4.37	66.6 ± 3.45	0.8209
Median [min, max]	66.0 [59.0, 77.0]	67.0 [59.0, 75.0]	
Weight			
Mean ± SD	155 ± 30.4	178 ± 41.5	0.0028
Median [min, max]	153 [102, 226]	169 [97.0, 260]	
Disease duration			
Mean ± SD	N/A	10.2 ± 8.73	N/A
Median [min, max]	N/A	7.07 [0.170, 33.9]	

*Note:* Biological data were compared using Welch’s t-tests unless otherwise noted.

**Table 2.** Sensorimotor data for healthy controls and people with multiple sclerosis.

	Healthy controls (HC)	Multiple sclerosis (MS)	p-value (HC vs. MS)
25 Foot walk (s)			
Mean ± SD	4.55 ± 0.527	5.79 ± 2.33	0.00377
Median [min, max]	4.55 [3.48, 5.66]	5.34 [3.53, 17.7]	
Sample size (n)	18	37	
Timed up and go (s)			
Mean ± SD	6.41 ± 0.825	8.48 ± 2.53	0.00004
Median [min, max]	6.31 [4.62, 7.6]	8.03 [5.01, 19.1]	
Sample size (n)	18	37	
Vibration sensation threshold			
Mean ± SD	1.34 ± 0.677	2.33 ± 1.32	0.00096
Median [min, max]	1.10 [0.7, 2.99]	1.83 [0.75, 5.32]	
Sample size (n)	18	33	
Bladder score			
Mean ± SD	3.42 ± 2.22	8.97 ± 7.21	0.00029
Median [min, max]	3.00 [0.00, 7.00]	7.00 [1.00, 27.0]	
Sample size (n)	19	31	
Expanded disability status scale (EDSS)	
Mean ± SD	N/A	1.72 ± 1.81	N/A
Median [min, max]	N/A	1.00 [0, 6.00]	
Sample size (n)	0	25	

*Note:* Sensorimotor data were compared using Welch’s t-tests unless otherwise noted.

### 2.3 Data acquisition

Sensorimotor testing included Timed Up and Go (Sebastião et al., 2016) (TUG), timed 25-foot walk (Inojosa et al., 2020) (T25w), and vibration threshold measurements for the great toe using a Vibratron-II device (Newsome et al., 2011). Two trials of the TUG and the T25w were recorded, and the average of these trials was calculated for both tests. Bilateral vibration detection thresholds were measured at the great toes using a descending method of limits, and the average of both feet was used in statistical analysis. Bladder symptoms were assessed using validated surveys for participant-reported outcome measures of bladder function based on the Lower Urinary Tract Dysfunction Research Network (LURN) Symptom Index 10 and 29 (Cella et al., 2020). These forms evaluate the severity and quality-of-life impact of urinary symptoms. A composite bladder score, calculated as the sum of scores from this LURN Symptom Index 10 and an additional 5 questions from LURN Symptom Index 29 (Supplementary Table S1), was used for analysis. This score ranges from 0 to 58, with higher values indicating more severe symptoms.

Imaging data were acquired for all participants using a 3T whole-body Philips dStream Ingenia MR scanner (Philips, Best, Netherlands), with an integrated 12-channel posterior SC array for reception and a dual-channel body coil for signal transmission. For spinal level registration and axial field of view positioning, a total-spine, sagittal T2-weighted turbo spin echo (TSE) sequence was acquired with SENSE = 1.4, TR/TE = 2765/120 ms, resolution = 1.0 × 1.1 mm^2^, slice thickness = 5 mm, number of slices = 11, field of view (FOV) = 270 × 270 mm^2^, and NSA = 2. Axial mFFE acquisitions optimized for the lower SC (O’Grady et al., 2023) were centered at the LSE and angled to maximize the number of slices perpendicular to the SC (Fig. 1), with parameters TR/TE1/ΔTE/α = 400 ms/6.4 ms/7.0 ms/43°, FOV = 150 x 150 mm^2^, 14 slices, 0.65 x 0.65 x 5 mm^3^ resolution, NSA = 3, magnetization transfer-weighted pre-pulse (16 ms duration, α = 600°, 5 kHz offset). A respiratory navigator was used for some participants when necessary to mitigate image degradation due to artifacts, but only one mFFE image volume per participant/scan session was included for statistical analysis. The sequence duration ranged from 8 minutes to 15 minutes, with the latter being due to the use of a respiratory navigator (O’Grady et al., 2023).

**Fig. 1.**
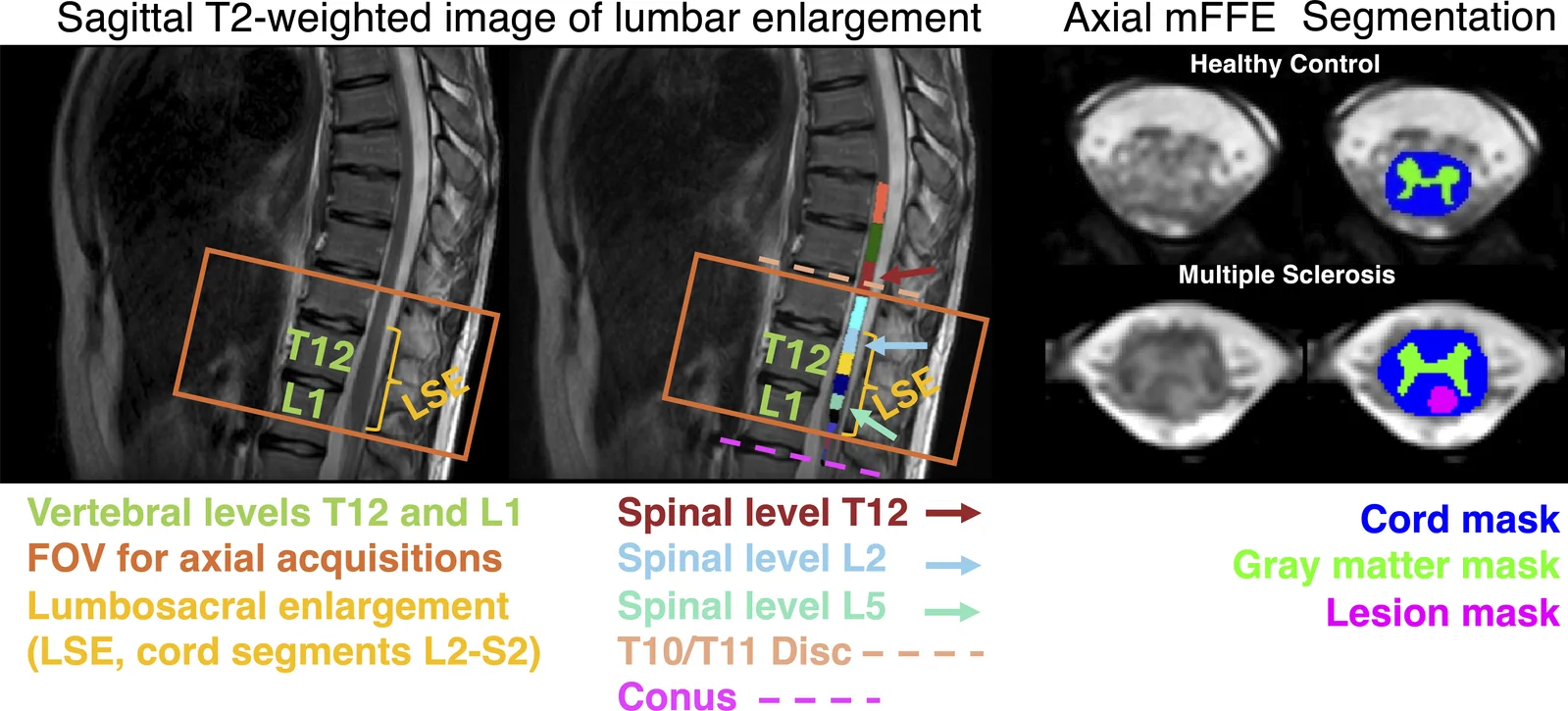
Left, center: The field of view (FOV) for the axial mFFE acquisition centered at the lumbosacral enlargement (LSE) and segmented spinal levels are shown overlaid on a sagittal T2-weighted image. Right: Examples of an axial mFFE slice in a healthy control (HC) and person with relapsing-remitting multiple sclerosis (pwRRMS). Segmentations of the LSE cord, gray matter, and lesion (pwRRMS only) are displayed. For most participants, the mFFE FOV spanned spinal levels T12, L1-L5, and S1-S4.

### 2.4 Image processing

Image processing was performed using FMRIB Software Library (FSL) v6.0.7.11 (Jenkinson et al., 2012) and Spinal Cord Toolbox (SCT) v6.2 (De Leener et al., 2017). The SC and GM were automatically segmented using SCT (*sct_deepseg_sc* and *sct_deepseg_gm*) and manually corrected, if necessary, prior to calculating slice-wise cord and GM CSA. In pwRRMS, lesions were delineated by C.D.M and S.S, with lesion masks drawn on the mFFE while referencing axial T2-weighted images (same acquisition geometry) as a clinical standard for each participant. A consensus lesion mask was generated for each participant with RRMS after review of the independently drawn masks. The Lesion:Cord CSA ratio normalizes lesion area to cord area through calculating the slice-wise ratio of lesion CSA to cord CSA; therefore, it represents the proportion of the cord impacted within the lumbosacral region studied and improves comparability across participants and spinal levels. Using SCT, template spinal levels were registered to the sagittal T2-weighted image using both the conus and T10/T11 intervertebral disc as landmarks (*sct_register_to_template*). For participants identified as outliers with respect to cord length and/or spinal level with maximal CSA (e.g. L1, S2) after the initial registration with template levels, the conus and C6/C7 intervertebral disc were used as landmarks to improve the template-based estimation of spinal levels in these cases. The sagittal T2 and mFFE were registered (*sct_register_multimodal*), and spinal levels were transformed into mFFE space (Fig. 1). Slice-wise cord, GM, and lesion CSA measurements were averaged within each spinal level for each participant. LSE length was estimated by multiplying the number of slices within spinal levels L2-S2 by 5 mm (slice thickness) for each participant.

### 2.5 Statistical analysis

We fit separate linear mixed-effects models (LMMs) on the morphometric measures (cord mean CSA, GM mean CSA, and mean GM:Cord CSA ratio for spinal levels T12 through S5) to evaluate overall effects of age, sex, height, weight, spinal level, and mFFE ‘type’ (+/- respiratory navigator) within HCs. We assumed a linear relationship for this mixed effect model because there are no evident nonlinear relationships between age, height, weight, and the CSA variables (Supplementary Fig. S1). Due to biological variability in SC length, spinal levels T9, T10, and T11 were covered by the mFFE FOV for some participants, but these levels were excluded from statistical analyses due to the small sample size. To assess spinal level-specific effects, we included interactions between spinal level and each biological variable in separate models. Spinal level was modeled as a categorical variable to allow level-specific estimates. To compare pwRRMS and HC, we fit separate LMMs on the morphometric measures with biological variables, group (HC or pwRRMS), and group-by-spinal-level interactions included as covariates, testing the interaction effect. To determine the effect of the Lesion:Cord CSA ratio on morphometric measurements, we fit LMMs on the outcomes of interest while adjusting for Lesion:Cord CSA ratio.

Lastly, we investigated the relationships between morphometric measures and measures of disability in pwRRMS. Due to sample size limitations, we restricted the analysis to spinal levels T12-S3 and further collapsed these levels into “upper” (T12-L4) and “lower” (L5-S3) regions of the LSE. We conducted six models with morphometric measures as the outcome measure and primary covariates of T25w, TUG, vibration sensitivity, bladder score, EDSS, or disease duration, with age, sex, and collapsed spinal levels (upper and lower) as additional covariates for each. Interaction terms between collapsed spinal levels and each primary covariate were included to assess region‐specific associations. Random intercepts for participant and visit nested in participant were included to account for repeated measurements within participants. We used an ordinal regression model (ORM) to assess the association between Lesion:Cord CSA ratio and disability measures. We fit a reduced ORM without the spinal level interaction term and extracted the p-value for the main effect including random effects for participant and the grouped spinal levels, after confirming that there were no significant interaction effects.

For all statistical tests, type 2 sum of squares in the car R package was used, so that main effects are tested without interaction terms in the model, and FDR (Benjamini-Hochberg) adjustment was applied across the four CSA measures as outcomes when testing the main effects and interactions. In models including spinal level–specific analyses, p-values obtained from tests performed at individual spinal levels were additionally adjusted for multiple comparisons across spinal levels. In this case, adjustment was performed separately within each outcome measure. All statistics were performed in R version 4.3.3. (R Foundation for Statistical Computing, Vienna, Austria).

## 3 Results

The MRI-derived measures of cord CSA, GM CSA, and GM:Cord CSA ratio for each spinal level in the LSE are summarized for HCs in Table 3, and the distribution of LSE length and spinal level with maximal CSA (e.g. L3, L4) are displayed in Supplementary Fig. S2). Results for modeling the overall effects of biological variables and mFFE “type” (with or without respiratory navigator) on cord and GM measures in HCs are presented in Table 4. No significant main effects survived FDR correction for any of the biological variables (age, height, weight, or sex) or mFFE type on any cord morphometric measures in HCs. When spinal level interaction effects (Supplementary Table S2) and relationships between biological variables and estimated marginal means of area measures were tested, none of the interaction effects were significant after FDR correction. However, for tests at individual spinal levels, age was significantly associated with GM CSA at spinal level L5 (p < 0.001, FDR-adjusted p-value (pFDR) = 0.003), and weight was significantly associated with the GM:Cord CSA ratio at spinal level S5 (p = 0.001, pFDR = 0.009) (Supplementary Fig. S3).

**Table 3.** MRI-derived measures of the cord cross-sectional area (CSA), gray matter (GM) CSA, and GM:cord CSA ratio in healthy controls.

Spinal level	Cord CSA (mm^2^)	GM CSA (mm^2^)	GM:cord CSA ratio
T12			
Mean ± SD	47.6 ± 6.18	9.20 ± 1.73	0.203 ± 0.0624
Median [min, max]	47.7 [29.3, 58.8]	8.99 [6.86, 12.8]	0.193 [0.126, 0.448]
L1			
Mean ± SD	49.2 ± 5.44	10.6 ± 2.30	0.215 ± 0.0377
Median [min, max]	49.0 [37.5, 62.0]	10.5 [6.39, 17.9]	0.213 [0.110, 0.324]
L2			
Mean ± SD	53.5 ± 6.60	12.8 ± 3.00	0.239 ± 0.0433
Median [min, max]	54.0 [39.7, 68.1]	12.2 [6.43, 23.5]	0.234 [0.140, 0.366]
L3			
Mean ± SD	57.1 ± 6.86	15.9 ± 3.33	0.279 ± 0.0542
Median [min, max]	57.5 [41.6, 70.7]	15.7 [7.97, 26.2]	0.272 [0.159, 0.440]
L4			
Mean ± SD	57.7 ± 7.56	17.5 ± 3.16	0.306 ± 0.0551
Median [min, max]	57.7 [38.6, 71.6]	17.2 [11.9, 25.4]	0.295 [0.171, 0.449]
L5			
Mean ± SD	54.0 ± 9.50	17.8 ± 3.78	0.333 ± 0.0646
Median [min, max]	55.9 [27.4, 72.0]	17.8 [7.55, 27.1]	0.332 [0.192, 0.535]
S1			
Mean ± SD	46.7 ± 11.0	16.0 ± 4.47	0.344 ± 0.0650
Median [min, max]	48.6 [15.9, 69.2]	15.7 [4.20, 24.5]	0.346 [0.0914, 0.519]
S2			
Mean ± SD	35.1 ± 11.1	12.4 ± 4.31	0.356 ± 0.0659
Median [min, max]	34.7 [13.5, 69.7]	12.0 [3.08, 23.9]	0.352 [0.144, 0.519]
S3			
Mean ± SD	25.8 ± 10.3	8.67 ± 4.06	0.336 ± 0.0781
Median [min, max]	24.6 [8.50, 59.8]	7.67 [3.16, 21.3]	0.337 [0.158, 0.605]
S4			
Mean ± SD	18.1 ± 7.95	6.51 ± 3.18	0.349 ± 0.0953
Median [min, max]	16.2 [5.22, 38.3]	5.66 [1.97, 15.9]	0.330 [0.177, 0.603]
S5			
Mean ± SD	15.0 ± 7.81	5.97 ± 2.90	0.348 ± 0.157
Median [min, max]	12.8 [2.90, 29.3]	5.14 [0.857, 9.78]	0.324 [0.119, 0.767]

**Table 4.** Linear mixed-effects model results and FDR adjusted p-values for overall effects of biological variables and mFFE type (+/- respiratory navigator) on cross-sectional area measures in the lumbosacral enlargement of healthy controls.

	Estimate	CI	p	p_FDR_
Cord CSA				
Age (years)	-0.109	-0.245 – 0.028	0.119	0.179
Height (in)	0.302	-0.402 – 1.006	0.399	0.598
Weight (lb)	-0.004	-0.076 – 0.068	0.909	0.909
Sex (male)	1.253	-4.234 – 6.739	0.654	0.867
mFFEtype (+nav)	1.515	-1.389 – 4.419	0.306	0.458
GM CSA				
Age (years)	-0.056	-0.103 – -0.010	0.017	0.052
Height (in)	-0.042	-0.281 – 0.197	0.731	0.731
Weight (lb)	0.003	-0.021 – 0.028	0.792	0.909
Sex (Male)	0.702	-1.130 – 2.533	0.452	0.867
mFFEtype (+nav)	0.688	-0.471 – 1.846	0.244	0.458
GM:cord CSA ratio				
Age (years)	-0.000	-0.001 – 0.001	0.452	0.452
Height (in)	-0.002	-0.007 – 0.003	0.371	0.598
Weight (lb)	0.000	-0.000 – 0.001	0.632	0.909
Sex (male)	0.003	-0.034 – 0.041	0.867	0.867
mFFEtype (+nav)	0.008	-0.016 – 0.033	0.507	0.507

Spinal level was also included as a fixed effect in these models, and random effects for participant and visit nested within participant were included.

The MRI-derived measures of cord CSA, GM CSA, GM:Cord CSA ratio, and Lesion:Cord CSA ratio for each spinal level are summarized for pwRRMS in Table 5. When controlling for all biological variables (age, sex, height, and weight), a significant main effect of group was observed for both cord CSA (p = 0.012, pFDR = 0.018, $\chi^{2}$ = 6.36, degrees of freedom (Df) = 1) and GM CSA (p = 0.011, pFDR = 0.018, $\chi^{2}$ = 6.52, Df = 1), indicating overall decreased morphometric measures in pwRRMS compared to HCs. Additionally, there were no significant interaction effects between spinal level and group for any morphometric measures (Supplementary Table S3). We performed individual FDR adjusted tests at each spinal level to identify what levels were driving the group differences. Group differences in cord CSA were detected at individual spinal levels L5 (p = 0.013, pFDR = 0.037), S1 (p = 0.002, pFDR = 0.027), S3 (p = 0.013, pFDR = 0.037), and S4 (p = 0.013, pFDR = 0.037) (Fig. 2A) and in GM CSA at spinal levels S1 (p = 0.003, pFDR = 0.029) (Fig. 2B) and S2 (p = 0.006, pFDR = 0.035). There were no significant group differences in GM:Cord CSA ratio at any individual spinal levels (Fig. 2C). Raw and FDR-adjusted p-values for marginal mean group differences are reported for all spinal levels in Supplementary Table S4.

**Fig. 2.**
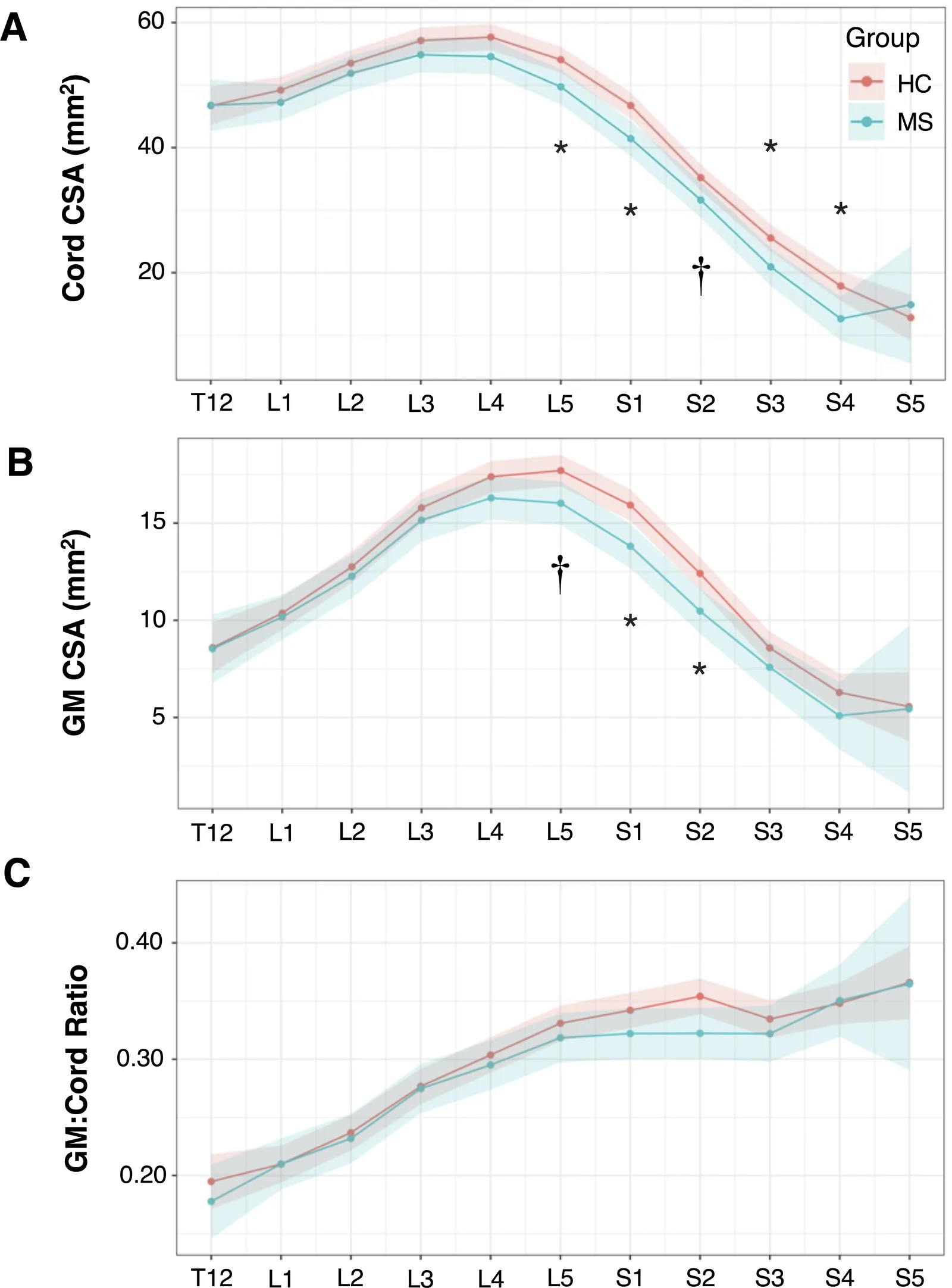
Marginal mean by group (HC and RRMS) in (A) Cord CSA, (B) GM CSA, and (C) GM:Cord CSA Ratio across all spinal levels (means adjusted for covariates). * indicates an FDR adjusted p-value < 0.05 and † indicates an FDR adjusted p-value < 0.1 for the marginal mean difference between HC and MS groups for a given spinal level.

**Table 5.** MRI-derived measures of the cord cross-sectional area (CSA), gray matter (GM) CSA, GM:cord CSA ratio, and lesion:cord CSA ratio in people with multiple sclerosis.

Spinal levels	Cord CSA (mm^2^)	GM CSA (mm^2^)	GM:cord CSA ratio	Lesion:cord CSA ratio
T12				
Mean ± SD	46.3 ± 4.24	8.71 ± 1.56	0.188 ± 0.0332	0.0449 ± 0.0598
Median [min, max]	47.1 [40.2,55.5]	8.43 [6.89,11.5]	0.189 [0.148,0.259]	0.0341 [0,0.200]
L1				
Mean ± SD	46.4 ± 5.74	9.97 ± 2.04	0.216 ± 0.0329	0.0706 ± 0.0986
Median [min, max]	47.0 [29.5,59.5]	10.1 [5.75,15.1]	0.212 [0.141,0.316]	0.0648 [0,0.568]
L2				
Mean ± SD	51.3 ± 7.07	12.1 ± 2.84	0.234 ± 0.0362	0.0911 ± 0.126
Median [min, max]	51.0 [37.7,67.6]	11.8 [6.69,20.2]	0.231 [0.173,0.317]	0.0643 [0,0.683]
L3				
Mean ± SD	54.1 ± 6.95	14.9 ± 3.10	0.276 ± 0.0474	0.0841 ± 0.111
Median [min, max]	54.6 [39.8,75.9]	14.9 [8.02,23.3]	0.271 [0.154,0.387]	0.0516 [0,0.552]
L4				
Mean ± SD	53.8 ± 6.38	16.1 ± 2.95	0.297 ± 0.0498	0.0927 ± 0.146
Median [min, max]	54.2 [38.7,67.3]	15.8 [10.0,23.9]	0.299 [0.156,0.393]	0.0508 [0,0.756]
L5				
Mean ± SD	49.0 ± 11.0	15.8 ± 3.56	0.320 ± 0.0625	0.0722 ± 0.160
Median [min, max]	50.5 [24.9,70.7]	16.2 [8.73,22.0]	0.323 [0.148,0.440]	0 [0,0.783]
S1				
Mean ± SD	40.7 ± 10.3	13.6 ± 3.71	0.324 ± 0.0613	0.0482 ± 0.155
Median [min, max]	40.4 [16.2,58.5]	13.8 [4.20,19.3]	0.335 [0.0967,0.432]	0 [0,0.782]
S2				
Mean ± SD	31.1 ± 9.58	10.4 ± 3.65	0.323 ± 0.0714	0.0341 ± 0.150
Median [min, max]	31.1 [10.0,49.9]	10.4 [3.59,17.6]	0.320 [0.193,0.446]	0 [0,0.933]
S3				
Mean ± SD	21.1 ± 7.94	7.48 ± 3.07	0.318 ± 0.0939	0.0263 ± 0.128
Median [min, max]	20.7 [3.50,35.4]	7.21 [3.35,14.2]	0.314 [0.0768,0.467]	0 [0,0.732]
S4				
Mean ± SD	13.5 ± 4.68	4.86 ± 1.43	0.348 ± 0.0647	0.0020 ± 0.009
Median [min, max]	13.9 [2.89,19.5]	5.15 [2.23,7.28]	0.353 [0.227,0.469]	0 [0,0.043]
S5				
Mean ± SD	12.6 ± 2.48	4.58 ± 0.443	0.368 ± 0.0373	0
Median [min, max]	12.6 [10.8,14.3]	4.58 [4.26,4.89]	0.368 [0.341,0.394]	0 [0,0]

To assess whether the group differences in morphometric measures are independent of the Lesion:Cord CSA ratio in pwRRMS, we repeated the analysis controlling for Lesion:Cord CSA ratio. This analysis showed that Lesion:Cord CSA ratio does influence observed group effects, which is evident through a slight reduction in the significance of the group effect on both cord (p = 0.012, pFDR = 0.018 with Lesion:Cord CSA ratio vs. p = 0.009, pFDR = 0.027 without) and GM CSA (p = 0.011, pFDR = 0.018 with Lesion:Cord CSA ratio vs. p = 0.019, pFDR = 0.028 without) compared to the analysis without Lesion:Cord CSA ratio, although the group differences in cord and GM CSA remain statistically significant (Supplementary Table S3).

We examined associations of clinical disability/sensorimotor measures (T25w, TUG, vibration sensitivity, bladder score, EDSS, and disease duration) with morphometric outcomes (cord CSA, GM CSA, GM:Cord CSA ratio) and Lesion:Cord CSA ratio in pwRRMS, grouping spinal levels into “upper” and “lower” bins. We did not find evidence for interaction effects across upper/lower spinal levels (Supplementary Table S5). There was a significant main effect between Lesion:Cord CSA ratio and average vibration sensation threshold (p < 0.001, pFDR < 0.001, $\chi^{2}$ = 21.645, Df = 1) (Fig. 3A) and EDSS (p < 0.001, pFDR < 0.001, $\chi^{2}$ = 13.816, Df = 1) (Fig. 3B) when controlling for age, sex, and spinal level (upper/lower). Finally, T25w, TUG, bladder score, and disease duration did not have any significant main effects. Main and interaction effects are reported for all disability metrics in Supplementary Table S5.

**Fig. 3.**
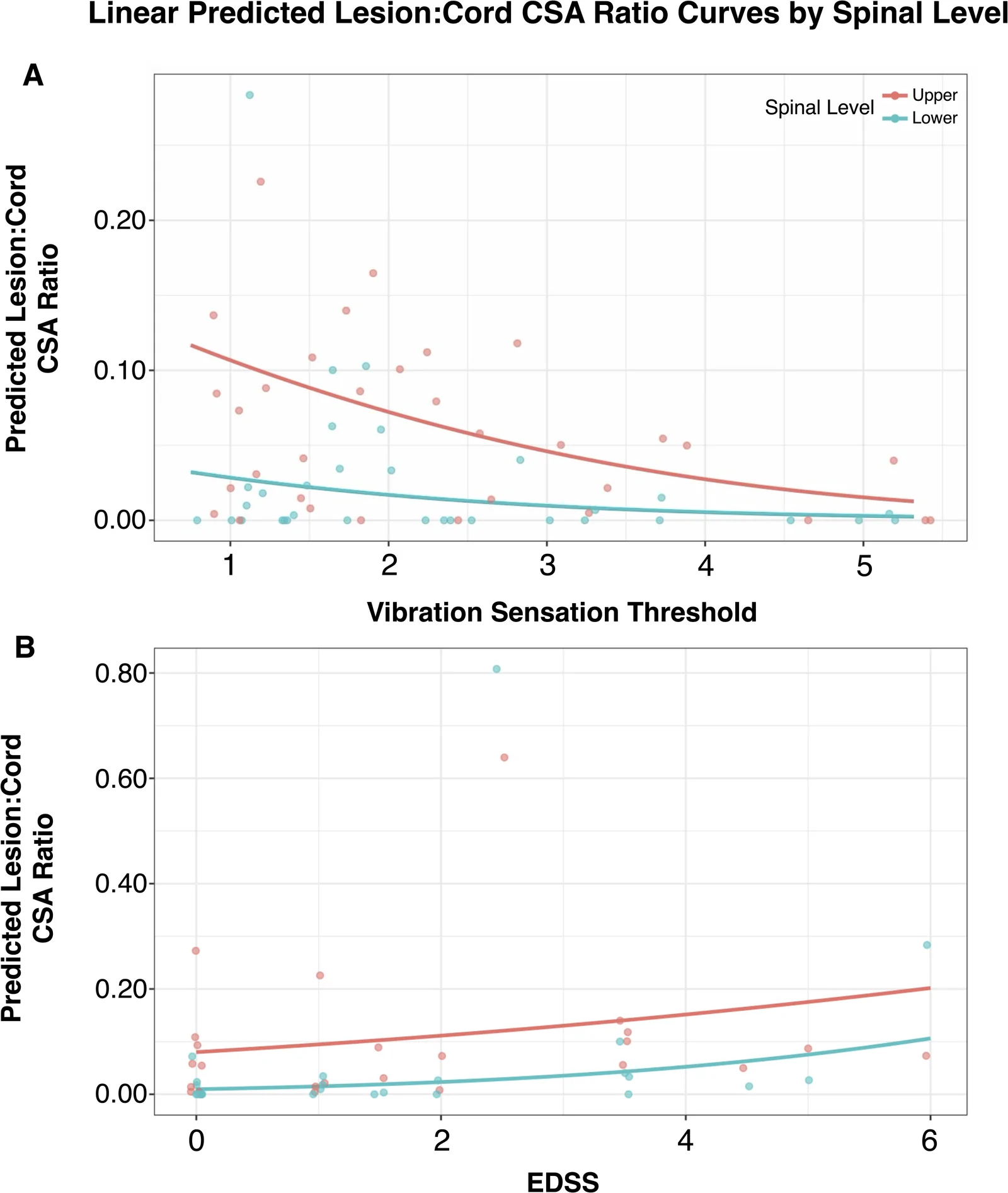
Predicted curves of Lesion:Cord CSA ratio for upper and lower spinal levels overlaid on scatterplots of averaged data points within each participant’s upper/lower spinal levels based on (A) vibration sensation threshold and (B) EDSS. A small amount of jitter was added for visualization, but the EDSS remains in increments of 0.5.

## 4 Discussion

In this study, we aimed to characterize the effects of age, sex, weight, and height on cord and GM CSA measures in the healthy LSE, examine disease-related changes in CSA measures in pwRRMS, determine the influence of Lesion:Cord CSA ratio on CSA measures, and correlate CSA and lesion measures with disability and sensorimotor metrics. All CSA values were derived from automated segmentation (with manual correction if necessary) of T2*-weighted mFFE images acquired at 3T in HCs and pwRRMS.

We found no significant main effects of age, height, weight, sex, or mFFE type on any CSA measures in HCs. However, we did observe a significant association between age and GM CSA at spinal level L5, and between weight and GM:Cord CSA ratio at spinal level S5. The estimated effect of weight on GM:Cord CSA ratio (Supplementary Fig. S3C) shows a larger 95% confidence interval at spinal level S5 compared to surrounding spinal levels, indicating that the precision of measurements at this level may be decreased. However, the 95% confidence interval at spinal level L5 for the estimated effect of age on GM CSA (Supplementary Fig. S3B) is comparable to surrounding spinal levels, suggesting increased confidence in this observation. The significant association with weight at the S5 spinal level could be possibly related to lower signal-to-noise ratio with greater weight affecting ability to segment the caudal tip of the cord. Additionally, the acquired image resolution, particularly the slice thickness of 5 mm, may cause some segmentation errors and/or partial volume effects for the most caudal spinal levels (i.e., S4, S5). Lastly, there was a significant main effect of age on GM CSA and a significant interaction between height and spinal level for GM CSA, but these did not remain significant after FDR correction. Therefore, we controlled for all biological variables in subsequent comparisons between HCs and pwRRMS to account for any differential effects.

In pwRRMS, both cord and GM CSA were reduced relative to HCs, and these reductions were influenced by Lesion:Cord CSA ratio. We also identified spinal level-dependent decreases in cord and GM CSA (Fig. 2), highlighting the importance of using an axial MRI sequence with sufficient resolution and GM contrast to facilitate studying GM changes in addition to overall SC atrophy. However, the increased variability in segmentation accuracy at the most caudal spinal levels, particularly for GM, may contribute to the lack of significant group differences at individual spinal levels such as S5. When correlating CSA measures to disability and sensorimotor/bladder function metrics, we found that T25w, TUG, bladder symptoms score, and disease duration, were not associated with any morphometric or Lesion:Cord CSA ratio outcomes. In contrast, average vibration sensation threshold and EDSS were associated with Lesion:Cord CSA ratio, but not with CSA measures. Vibration sensation threshold was negatively associated while EDSS was positively associated with predicted Lesion:Cord CSA ratio. While higher (worse) EDSS score is expected with increasing Lesion:Cord CSA ratio, a decreasing (better) vibration sensation threshold with increasing Lesion:Cord CSA ratio is not expected. This unexpected negative association between Lesion:Cord CSA ratio and vibration threshold may be explained by the limited sample size with very few pwRRMS having no lesions in the LSE in our cohort. Additionally, this study does not account for any added lesions in the brain and upper spinal levels, which may also affect sensory function as measured by the vibration test in the great toe.

Previous studies of SC morphometry using MRI have reported variable associations between SC measurements of size (e.g., CSA or volume) and biological variables. Sex is a common and consistently reported factor influencing SC morphometry. Several studies have reported significant correlations between sex and SC measurements, with females generally exhibiting smaller CSA in the cervical cord (Cook et al., 2024; Kesenheimer et al., 2021; Papinutto et al., 2015, 2019; Ulbrich et al., 2014; Valošek et al., 2024). However, these differences have not been significant in the thoracic cord (Kesenheimer et al., 2021; Papinutto et al., 2015). The findings in the LSE are mixed, with one study finding greater GM CSA in men but no sex difference in cord CSA (Kesenheimer et al., 2021), while another study found no significant sex-related differences in cord or GM volumes (Yiannakas et al., 2019). Our results align with the latter, as we observed no significant sex effects in the LSE. We could attribute this finding to several factors: the sample size and sex distribution may be insufficient and/or the current image resolution may limit detection of small differences, or there may be a true biological absence or much weaker influence of sex differences on SC morphometry at lumbosacral levels. If the latter is true, a possible explanation for this regional difference is that the cervical cord’s proximity to the brain, an area known to show structural changes with age and sex, may cause demographic-related changes to propagate more strongly into proximal cervical cord regions than into more distant lumbosacral segments. Supporting this hypothesis, previous work demonstrates correlations between cervical and thoracic cord morphometrics and brain tissue volumes (Papinutto et al., 2015), and another study found sex effects in cervical and thoracic cord but not in the lumbar enlargement (Joo et al., 2025); additionally, post-mortem analysis suggests lumbar cord length is less variable across individuals than more proximal segments (Glaudot et al., 2025).

Age-related effects on SC morphometrics also vary by region. Multiple studies report a decrease in cervical cord, GM, and WM CSA with increasing age depending on the cervical vertebral level (Cook et al., 2024; Kesenheimer et al., 2021; Papinutto et al., 2015), though some found only moderate or no correlations (Labounek et al., 2025; Papinutto et al., 2019; Ulbrich et al., 2014). The thoracic cord also has varying effects of age between studies, with one finding decreases in thoracic cord, GM, and WM CSA with age (Papinutto et al., 2015), while another study found a significant difference in thoracic GM CSA between young and middle-aged volunteers as well as thoracic total cord CSA between middle-aged and older volunteers (Kesenheimer et al., 2021). In contrast, LSE studies, including ours, generally report no significant relationship between age and cord or GM CSA or volumes (Kesenheimer et al., 2021; Yiannakas et al., 2019).

Height has shown inconsistent correlations with SC morphometric measures. Some studies report no significant associations with cervical cord, GM, or WM CSA (Papinutto et al., 2015), while others found correlations with GM:Cord CSA ratio at certain cervical levels (Cook et al., 2024) or primarily with cord and WM more than GM (Labounek et al., 2025). Weight has generally shown little to no consistent association with SC morphometrics across cervical (Cook et al., 2024; Labounek et al., 2025; Papinutto et al., 2015), thoracic (Papinutto et al., 2015), and LSE (Kesenheimer et al., 2021) levels, consistent with our finding of no main effects of weight.

Previous studies have quantified LSE morphometrics using different MRI sequences and definitions of the enlargement. Many defined the LSE as the slice with maximal CSA, averaging across 1–3 neighboring slices. Two studies using axial 2D radially sampled averaged magnetization inversion recovery acquisitions (rAMIRA) reported mean cord CSA of 62.54 ± 6.93 mm^2^ (Wendebourg et al., 2024) and 62.9 ± 6.5 mm^2^ (Kesenheimer et al., 2021) with corresponding mean GM CSA of 25.28 ± 3.33 mm^2^ (Wendebourg et al., 2024) and 24.0 ± 3.8 mm^2^ (Kesenheimer et al., 2021). Two studies using a 3D slab-selective fast field echo (FFE) sequence reported mean cord CSA 62.3 ± 4.1 mm^2^ (Yiannakas et al., 2014) and 59.3 ± 8.4 mm^2^ (Ugorji et al., 2015) with mean GM CSA of 19.8 ± 3.3 mm^2^ (Yiannakas et al., 2014) and 17.0 ± 3.1 mm^2^ (Ugorji et al., 2015). Two studies using an axial T2*-weighted 3D spoiled multi-echo gradient-echo sequence reported mean cord CSA of 60.3 ± 6.6 mm^2^ (Büeler et al., 2024) and 69.2 ± 7.1 mm^2^ (David et al., 2022) with corresponding mean GM CSA 27.5 ± 3.9 mm^2^ (Büeler et al., 2024) and 21.1 ± 2.6 mm^2^ (David et al., 2022). Using a comparable axial T2*-weighted mFFE sequence, we identified spinal level L4 as having the largest CSA (Table 3, Supplementary Fig. S2B) with mean LSE cord CSA of 57.7 ± 7.56 mm^2^ and GM CSA of 17.5 ± 3.16 mm^2^. Additionally, previous postmortem studies have also identified spinal level L4 as the level with the largest CSA (Kameyama et al., 1996; Ko et al., 2004). While the cord CSA values are similar across these studies, there is greater variability in measurements of GM CSA which could be due to differences in the acquired voxel size of sequences used, segmentation methods, and age/sex distributions of cohorts although we don’t expect significant biological variability. For example, the larger GM CSA in (Kesenheimer et al., 2021) was derived from a sequence with 8 mm slice thickness, while our measurement and the similar GM CSA measurement in Ugorji et al. (2015) were derived from acquisitions with 5 mm slice thickness.

To our knowledge, only one publication (Bussas et al., 2022) and one pilot study (Liechti et al., 2017) have measured cord and/or GM CSA in the LSE of pwMS to date. The published study used a 2D T2-weighted TSE sequence and reported mean cord CSA in pwRRMS of ~52 mm^2^ compared to ~55 mm^2^ in HCs at vertebral level T11, identified as the maximum of the lumbar enlargement (Bussas et al., 2022). However, this study did not investigate GM morphometrics, since T2-weighted images do not create sufficient GM:WM contrast, further highlighting the benefits of T2*-weighted images in the LSE. The pilot study used a 3D slab-selective FFE sequence and reported a cord CSA of 55.9 ± 6.5 mm^2^ and GM CSA of 20.0 ± 3.3 mm^2^ in pwMS, compared to 59.6 ± 6.5 mm^2^ and GM CSA as 23.4 ± 3.8 mm^2^ in HCs (Liechti et al., 2017). Similar to our findings in the present study, both of these studies found significant differences in CSA measures between HC and pwMS, however these were not homogeneous. Bussas et al. reported a significant difference between HC and pwMS cord CSA at vertebral level T11 as well as adjacent vertebral levels (Bussas et al., 2022). However, Liechti et al. found a significant difference between HC and pwMS for LSE GM CSA but not cord CSA (Liechti et al., 2017).

Beyond these few studies, evidence for MS-related atrophy in the LSE remains limited. One article assessed cord area across the entire cord, reporting smaller mean CSA in pwRRMS compared to HCs with coverage from cervical levels to the conus medullaris (Bussas et al., 2022). In the lower thoracic cord, one study found that pwRRMS demonstrated selective GM atrophy and that people with primary progressive MS (pwPPMS) showed more substantial cord and GM CSA reductions than pwRRMS (Schlaeger et al., 2015). Another study spanning cervical and thoracic regions found cord CSA reductions in pwRRMS at cervical but not thoracic levels (Mina et al., 2021). Together, these findings suggest that MS-related atrophy differs by spinal region and disease subtype, underscoring the need for region-specific investigations.

Correlations between cord morphometry and disability also vary across studies. Lower thoracic GM CSA has been associated with both disability and MS subtype (PPMS vs. RRMS), supporting the role of GM loss as a key mediator of MS disability (Schlaeger et al., 2015). In a postmortem 7T MRI study assessing cervical SC lesion burden and CSA across RRMS, SPMS, and PPMS, both T2* hyperintensity burden and CSA correlated with EDSS, with cases of greatest disability showing significantly greater lesion burden than those with least disability (Mahajan et al., 2025). Additionally, an in vivo cervical SC MRI study found negative correlations between EDSS and lower cervical CSA but no significant relationship between disease duration and cervical CSA, the latter consistent with findings in our cohort (Celik et al., 2023). In a study assessing morphometrics in the entire cord from cervical to the conus, RRMS EDSS scores did not correlate with SC volumes but did correlate with lesion volume (Bussas et al., 2022). The authors suggested that compared to cord lesions that contribute to clinical disability in both pwRRMS and pwPPMS, cord atrophy becomes detectable early in pwRRMS but contributes to disability only later in the disease course (Bussas et al., 2022). This may help explain the lack of significant associations between morphometrics and disease metrics in our cohort. That study also examined the contribution of lesions and initially reported no interplay between cord atrophy and lesion volume. However, this was considered an unexpected finding, as pwMS with the largest cord lesion volumes did not differ significantly from those without lesions (Bussas et al., 2022). In contrast, our results demonstrated a partial mediation of the effect of disease on cord/GM CSA by Lesion:Cord CSA ratio, suggesting that this relationship requires further study. Finally, they noted that the association between EDSS and SC atrophy diminished in more caudal regions (Bussas et al., 2022), aligning with our observation that LSE morphometrics were not significantly associated with EDSS. However, EDSS scores from routine clinic visits were not available for a subset of the pwRRMS in this study which is a limitation of the EDSS association analysis.

With this, we recognize additional limitations of our study. First, our pwRRMS cohort primarily included participants with relatively low levels of disability. Future work could extend this study to secondary and primary progressive MS, where greater atrophy and stronger clinical correlations may be detectable as demonstrated in a previous study (Bussas et al., 2022). Second, the age range for this study had a maximum age of ~64 years in the pwRRMS group, limiting our ability to evaluate age-related changes across the lifespan. Third, the observation of fewer lesions in the lower (sacral) spinal levels could be due to limitations on visibility with the acquired mFFE resolution rather than true absence of lesions. Additionally, the template-based registration using anatomical landmarks remains a limitation in this study, and future work could improve image resolution to visualize nerve roots and enable spinal level assignment directly in native images for individual participants. Finally, as the focus of this study was on applying an optimized anatomical sequence in the LSE of pwRRMS, the correlations with disability do not consider possible contributions from lesions or atrophy upstream in the upper (cervical and thoracic) spinal levels or the brain.

In conclusion, this study demonstrates that segmentation of axial T2*-weighted mFFE images optimized for intracord tissue (WM/GM) and lesion contrast can quantify cord and GM morphometry as well as Lesion:Cord CSA ratio in the LSE. We show that pwRRMS exhibit significant reductions in both cord and GM CSA, partially influenced by Lesion:Cord CSA ratio, and that there are spinal level-dependent decreases in GM CSA and GM:Cord CSA ratio. While CSA did not correlate with disability, bladder symptoms, or sensorimotor metrics, lesion presence was associated with EDSS scores and vibration sensitivity, highlighting differential contributions of atrophy and lesions to disability in this cohort of pwRRMS. The detection of cord and GM atrophy in the LSE confirms that this region is impacted by disease and highlights the importance of incorporating GM-sensitive, region-specific spinal imaging into future studies to better define MRI-derived biomarkers and their relationship to clinical outcomes.

## Supplementary Material

Supplementary Material

## Data Availability

Access to the human MRI data for this study are subject to the policies of Vanderbilt University Medical Center. Requests for a formal data sharing or data use agreement and analysis code for research purposes may be directed to the corresponding author. The relevant software to perform spinal cord segmentation and quantification of morphometry is publicly available through the Spinal Cord Toolbox (https://spinalcordtoolbox.com/stable/) and FMRIB Software Library (https://fsl.fmrib.ox.ac.uk/fsl/docs/index.html) websites.
