# Supplementary material for: Optimized multi-echo gradient echo MRI of the lumbosacral spinal cord to investigate atrophy, lesions, and disability in relapsing-remitting multiple sclerosis
Source: Imaging Neurosci (Camb). 2026 Nov 10;4:IMAG.a.1410. doi: 10.1162/IMAG.a.1410 (PMC13754069; doi:10.1162/IMAG.a.1410)
Supplement: Supplementary file 1 [file IMAG.a.1410_supp.pdf]

Supplementary Materials

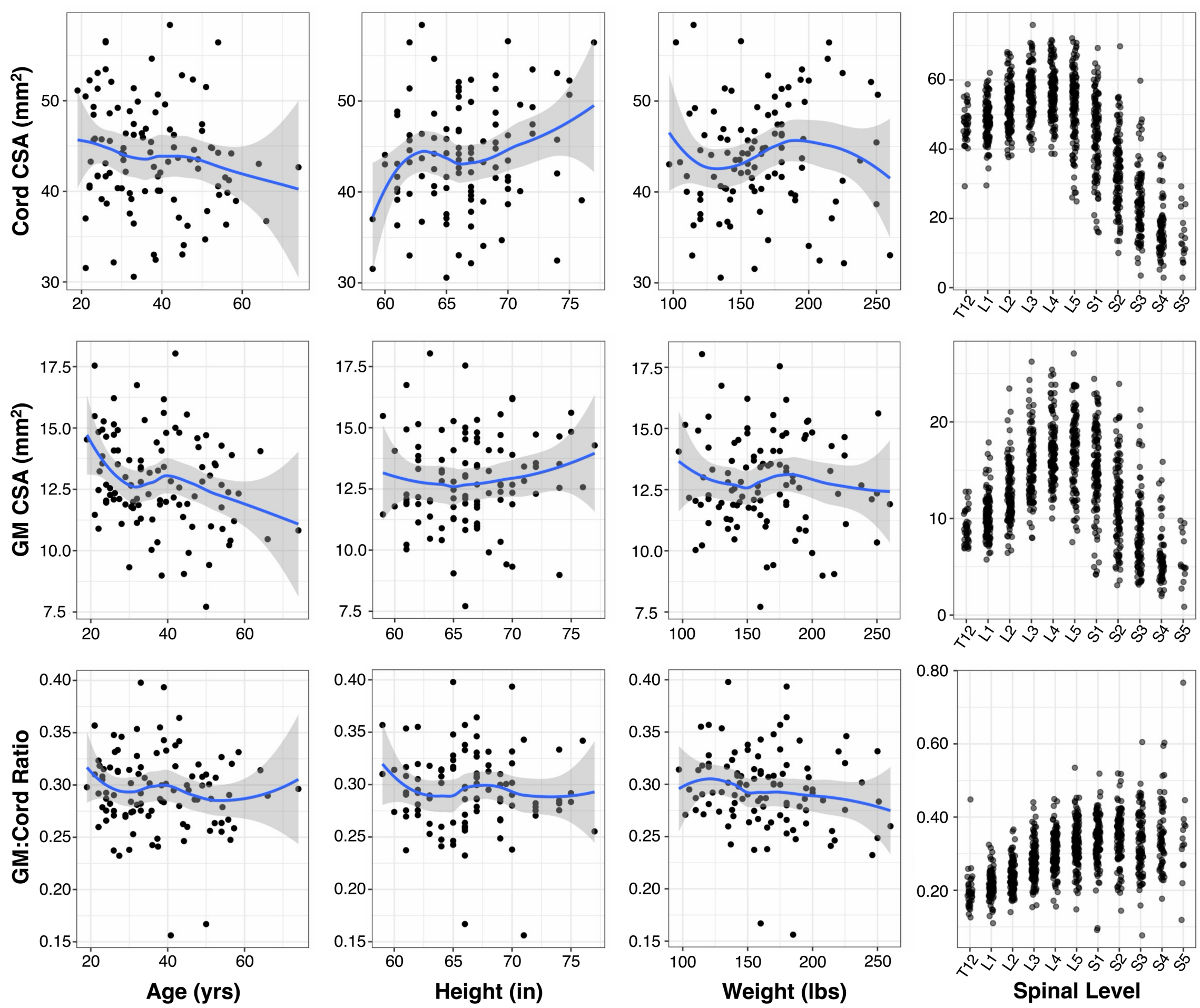

Supplementary Figure 1. Scatter plots for age, height, and weight are based on participant-level data, with morphometric measures averaged across spinal levels within each healthy control. Spinal-level plots (right) include all available spinal-level observations. Blue curves represent locally estimated scatterplot smoothing (LOESS) curves with 95% confidence intervals.

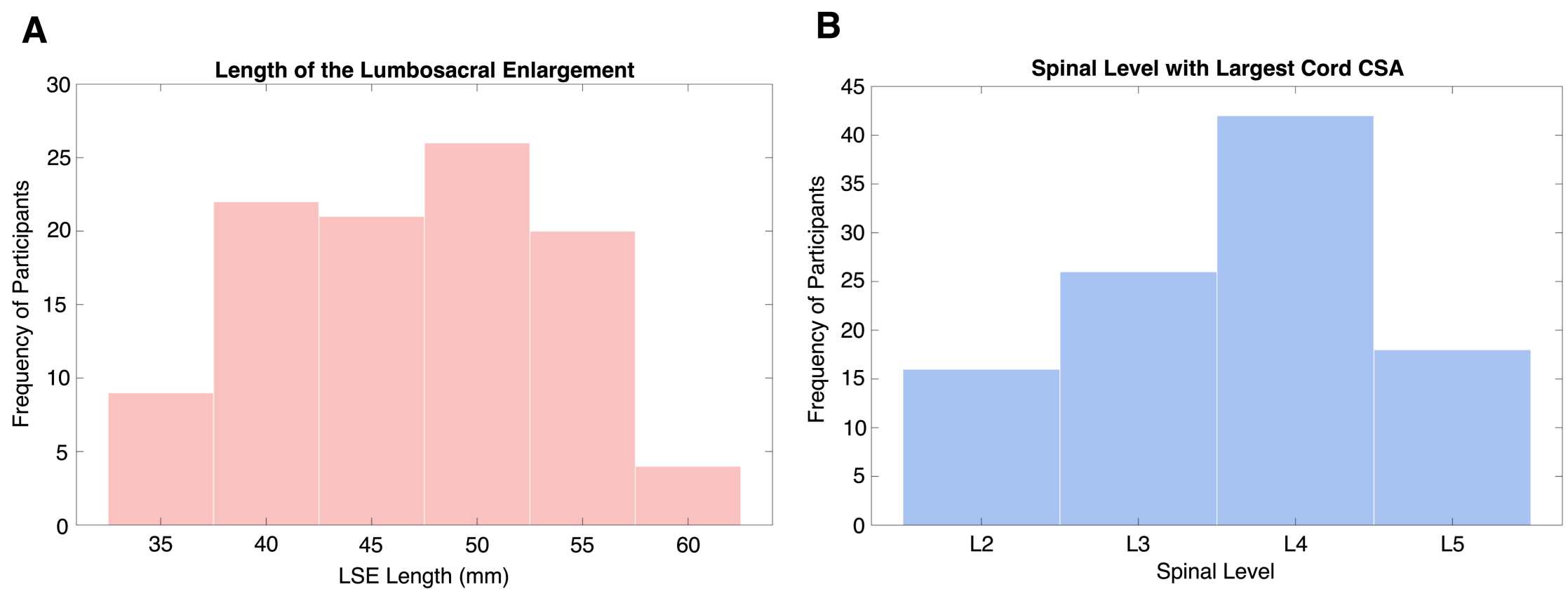

Supplementary Figure 2. Distributions of **A)** estimated length of the lumbosacral enlargement (LSE) in mm and **B)** spinal level with the largest cord cross-sectional area (CSA) across all healthy controls (HC) (n=60) and people with relapsing-remitting MS (pwRRMS) (n=42).

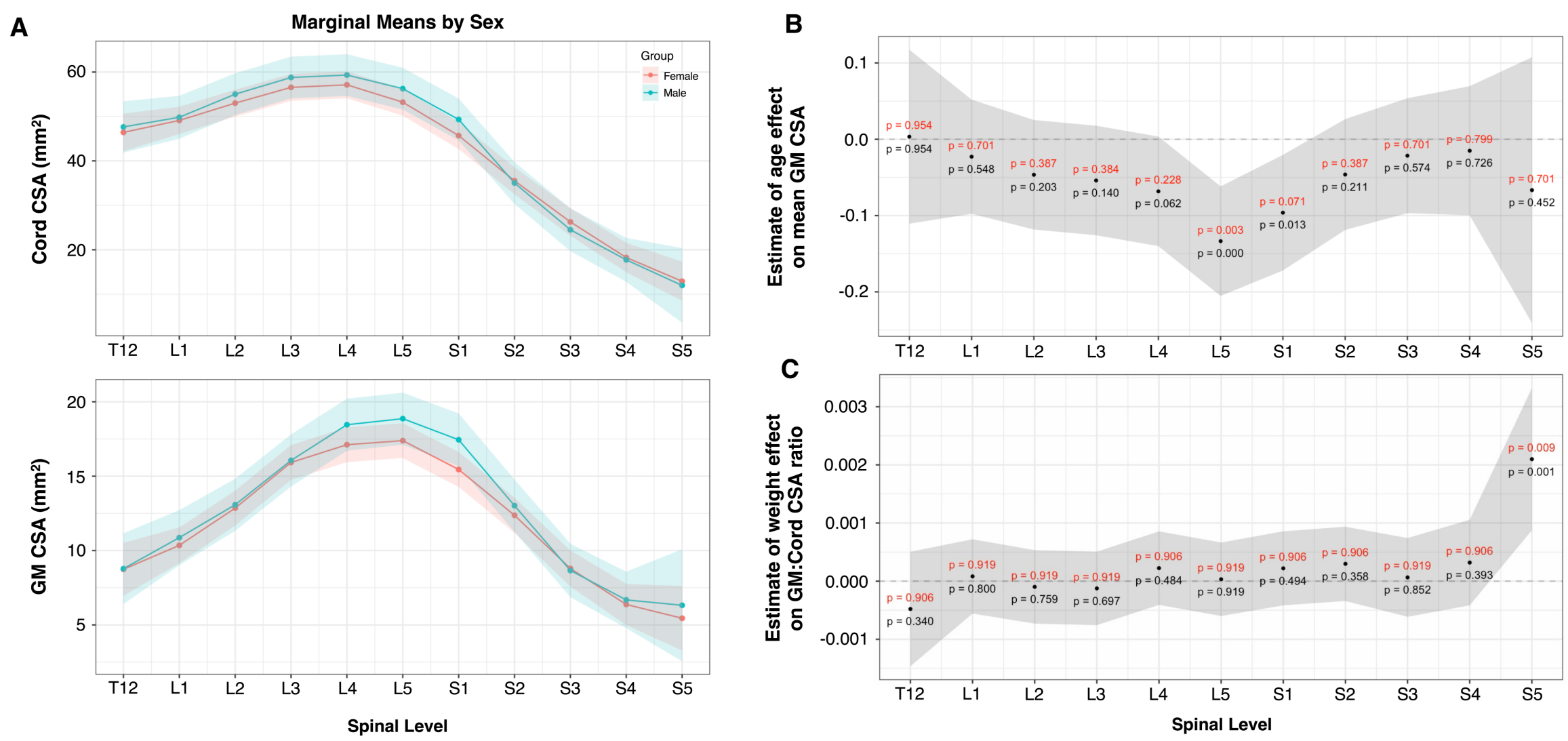

Supplementary Figure 3: A) Marginal mean by sex in cord and gray matter (GM) cross-sectional area (CSA), **B)** Estimated effect of age on average GM CSA and **C)** Estimated effect of body weight on GM:Cord CSA ratio across all spinal levels (T12-S5) in healthy controls (n=60). **B and C)** Each point represents estimated slopes at each spinal level and shaded areas represent 95% confidence intervals. Black p-values represent unadjusted values; red p-values are FDR-adjusted.

Supplementary Table 1: Bladder symptoms survey administered to participants, including LURN Symptom Index-10 (LURN SI-10) questions and 5 additional questions from LURN Symptom Index-29 (LURN SI-29). Instruction given to participant: "This questionnaire asks you about different urinary symptoms. Please read each question carefully, and then select the response that best describes your symptoms."

LURN Symptom Index	Question	**Never**	**A few times**	**About half the time**	**Most of the time**	**Every time**
LURN 10	In the past 7 days, how often did you feel a sudden need to urinate?	0	1	2	3	4
LURN 10	In the past 7 days, how often did you leak urine or wet a pad after feeling a sudden need to urinate?	0	1	2	3	4
LURN 10	In the past 7 days, how often did you leak urine or wet a pad while laughing, sneezing, or coughing?	0	1	2	3	4
LURN 10	In the past 7 days, how often did you leak urine or wet a pad when doing physical activities, such as exercising or lifting a heavy object?	0	1	2	3	4
LURN 10	In the past 7 days, how often did you have pain or discomfort in your bladder while it was filling?	0	1	2	3	4
LURN 10	In the past 7 days, how often did you have a delay before you started to urinate?	0	1	2	3	4
LURN 10	In the past 7 days, how often was your urine flow slow or weak?	0	1	2	3	4
LURN 10	In the past 7 days, how often did you dribble urine just after zipping your pants or pulling up your underwear?	0	1	2	3	4
LURN 10	In the past 7 days, during waking hours, how many times did you typically urinate?	0 (3 or fewer times a day)	1 (4-7 times a day)	2 (8-10 times a day)	3 (11 or more times a day)	
LURN 10	In the past 7 days, during a typical night, how many times did you wake up and urinate?	0 (none)	1 (1 time)	2 (2-3 times)	3 (More than 3 times)	

LURN 29	In the past 7 days, how often did you leak urine during the night, including wetting a pad or the bed?	0	1	2	3	4
LURN 29	In the past 7 days, how often did you have pain or discomfort while urinating?	0	1	2	3	4
LURN 29	In the past 7 days, how often did you have to push when urinating?	0	1	2	3	4
LURN 29	In the past 7 days, once you started urinating, how often did your urine flow stop and start again?	0	1	2	3	4
LURN 29	In the past 7 days, how often did you feel that your bladder was not completely empty after urination?	0	1	2	3	4

Supplementary Table 2: Interaction effect between sex, age, height, and weight and spinal levels on all cross-sectional area measures for cord and gray matter in healthy controls.

	Interaction Effect			
	χ^2	DF	p	p_{FDR}
**Sex**				
Cord CSA	10.49	10	0.399	0.806
GM CSA	7.50	10	0.677	0.806
GM:Cord CSA	6.11	10	0.806	0.806
**Age**				
Cord CSA	14.4	10	0.155	0.184
GM CSA	13.8	10	0.184	0.184
GM:Cord CSA	17.0	10	0.074	0.184
**Height**				
Cord CSA	6.25	10	0.793	0.793
GM CSA	12.02	10	0.284	0.793
GM:Cord CSA	8.83	10	0.548	0.793
**Weight**				
Cord CSA	6.09	10	0.807	0.807
GM CSA	8.42	10	0.588	0.807
GM:Cord CSA	18.70	10	0.044	0.132

GM = gray matter, WM = white matter, CSA = cross-sectional area, GM:Cord CSA = GM to Cord CSA ratio, DF = degree of freedom

Supplementary Table 3. Main and Interaction effect between group (healthy control vs. multiple sclerosis) and spinal levels on all cross-sectional area measures for cord and gray matter.

	Main Effect				Interaction Effect			
	χ^2	DF	p	p_{FDR}	χ^2	DF	p	p_{FDR}
**Group Difference**								
Cord CSA	6.36	1	0.012	0.018	12.67	10	0.243	0.509
GM CSA	6.52	1	0.011	0.018	11.24	10	0.339	0.509
GM:Cord CSA	1.44	1	0.230	0.230	8.33	10	0.597	0.597
**Group Difference Controlled for Lesion:Cord CSA Ratio**								
Cord CSA	6.834	1	0.009	0.027	-	-	-	-
GM CSA	5.525	1	0.019	0.028	-	-	-	-
GM:Cord CSA	0.864	1	0.353	0.353	-	-	-	-

GM = gray matter, WM = white matter, CSA = cross-sectional area, GM:Cord CSA = GM to Cord CSA ratio, DF = degree of freedom

Supplementary Table 4: Unadjusted and FDR-adjusted p-values (correction with Benjamini-Hochberg method) for the marginal mean group differences between healthy controls and people with relapsing-remitting multiple sclerosis for all spinal levels and cross-sectional area measures.

		Spinal Level										
		**T12**	**L1**	**L2**	**L3**	**L4**	**L5**	**S1**	**S2**	**S3**	**S4**	**S5**
**Cord CSA**												
MS-HC	p	0.979	0.271	0.353	0.190	0.072	0.013	0.002	0.043	0.013	0.013	0.686
	p_{FDR}	0.979	0.372	0.431	0.298	0.131	0.037	0.027	0.094	0.037	0.037	0.754
**GM CSA**												
MS-HC	p	0.959	0.775	0.479	0.352	0.113	0.015	0.003	0.006	0.203	0.231	0.960
	p_{FDR}	0.960	0.947	0.659	0.553	0.311	0.055	0.029	0.035	0.423	0.423	0.960
**GM:Cord CSA**												
MS-HC	p	0.393	0.995	0.701	0.881	0.508	0.337	0.130	0.017	0.384	0.899	0.976
	p_{FDR}	0.864	0.995	0.995	0.995	0.932	0.864	0.715	0.189	0.864	0.995	0.995

GM = gray matter; CSA = cross-sectional area; MS = multiple sclerosis; HC = healthy control

Supplementary Table 5: Main and interaction (between disability metrics and spinal levels upper/lower) effects on all cross-sectional area measures for cord and gray matter and lesion measures.

	Main Effect				Interaction Effect			
	χ^2	DF	p	p_{FDR}	χ^2	DF	p	p_{FDR}
**Average 25 Foot Walk**								
Cord CSA	0.167	1	0.682	0.682	0.061	1	0.804	0.838
GM CSA	2.331	1	0.127	0.254	0.042	1	0.838	0.838
GM:Cord CSA	0.378	1	0.538	0.682	0.422	1	0.516	0.838
Lesion:Cord CSA	2.419	1	0.120	0.254	1.860	1	0.395	0.838
**Average Up and Go**								
Cord CSA	0.033	1	0.856	0.856	0.033	1	0.855	0.938
GM CSA	1.643	1	0.200	0.536	0.006	1	0.938	0.938
GM:Cord CSA	0.184	1	0.668	0.856	0.584	1	0.445	0.938
Lesion:Cord CSA	1.229	1	0.268	0.536	0.658	2	0.720	0.938
**Average Vibration Threshold**								
Cord CSA	1.183	1	0.277	0.485	2.308	1	0.129	0.516
GM CSA	0.761	1	0.383	0.485	0.911	1	0.340	0.680
GM:Cord CSA	0.487	1	0.485	0.485	0.107	1	0.744	0.930
Lesion:Cord CSA	21.645	1	<0.001	<0.001	0.146	2	0.930	0.930
**Bladder Score**								
Cord CSA	0.046	1	0.830	0.830	0.590	1	0.442	0.589
GM CSA	1.553	1	0.213	0.365	2.357	1	0.125	0.250
GM:Cord CSA	1.197	1	0.274	0.365	3.805	1	0.051	0.204
Lesion:Cord CSA	2.339	1	0.126	0.365	0.475	2	0.788	0.788
**EDSS**								
Cord CSA	0.000	1	0.989	0.989	1.381	1	0.240	0.336
GM CSA	1.776	1	0.183	0.366	0.558	1	0.455	0.455
GM:Cord CSA	0.245	1	0.621	0.828	1.313	1	0.252	0.336
Lesion:Cord CSA	13.816	1	<0.001	<0.001	5.975	2	0.050	0.200
**Disease Duration**								
Cord CSA	0.151	1	0.698	0.859	0.585	1	0.444	0.679
GM CSA	1.044	1	0.307	0.614	0.058	1	0.810	0.810
GM:Cord CSA	3.174	1	0.075	0.300	0.436	1	0.509	0.679
Lesion:Cord CSA	0.031	1	0.859	0.859	1.408	2	0.495	0.679

GM = gray matter, WM = white matter, CSA = cross-sectional area, GM:Cord CSA = GM to Cord CSA ratio, Lesion:Cord CSA = Lesion to Cord CSA ratio, DF = degree of freedom
